# Symptomatic Recovery from Concussion in Military Service Members with and Without Associated Bodily Injuries

**DOI:** 10.1089/neur.2024.0041

**Published:** 2024-08-22

**Authors:** Jan Elizabeth Kennedy, Joseph Booth Warren, Lisa Hsiao-Jung Lu, Cristina Yvette Lawrence, Matthew Wade Reid

**Affiliations:** ^1^ Contractor, General Dynamics Information Technology (GDIT), Falls Church, Virginia, USA.; ^2^ Traumatic Brain Injury Center of Excellence (TBICoE), Research Portfolio Management Division, Research & Engineering Directorate, Defense Health Agency, Falls Church, Virginia, USA.; ^3^ Brooke Army Medical Center, Neurology Service (MCHE-ZDM-N), JBSA Fort Sam Houston, Fort Sam Houston, Texas, USA.; ^4^ Contractor, CICONIX, LLC, Annapolis, Maryland, USA.

**Keywords:** bodily injury, mild TBI, military, pain, social support, symptomatic outcome

## Abstract

Research has found that service members (SMs) with mild traumatic brain injury (mTBI) and co-occurring bodily injuries endorse lower chronic postconcussive symptom severity than SMs with mTBI and no bodily injuries. Investigations were conducted with primarily post–9/11 war-era SMs with blast injuries. The current study explores these findings in a cohort of more heterogeneous and recently evaluated military SM. Possible reasons suggested for the earlier findings include SMs with bodily injuries report fewer postconcussive symptoms due to (1) focusing attention on extra-cranial injuries and associated pain; (2) receiving more interpersonal and medical support, lowering distress; (3) using analgesics such as morphine or opioids; or (4) experiencing delayed postconcussive symptoms. The current investigation evaluates each of these hypothesized reasons for the earlier findings and the generalizability of the findings to a more recent sample. Data were extracted from 165 SMs in a TBI repository at a U.S. military medical center. All participants reported a history of an mTBI, confirmed by a clinical interview to meet Veterans Affairs and Department of Defense criteria. Other bodily injuries received at the time of the mTBI were documented with the Abbreviated Injury Scale (AIS). Multiple regression models evaluated the ability of the four hypothesized mechanisms to predict postconcussive symptom severity, measured by the Neurobehavioral Symptom Inventory. SMs with bodily injuries (*n* = 48) reported nonsignificantly lower postconcussive symptoms than SMs with no bodily injuries (*n* = 117). The level of subjective pain was a determinant of postconcussive symptom severity among SMs with a history of mTBI, with or without associated bodily injuries. Social support was a weaker negative predictor of postconcussive symptoms among SMs with no associated bodily injuries.

## Introduction

Traumatic brain injury (TBI) as defined by the U.S. Military Health System is a “traumatically induced structural injury of the brain or physiologic disruption of normal brain function resulting from an external force.”^1^ Patients with TBI may show a combination of acute signs and symptoms, including periods of lost or altered levels of consciousness or amnesia, as well as neurological deficits and/or intracranial lesions on imaging.^2^ From 2000 through the fourth quarter of 2022, 472,785 U.S. service members (SMs) were diagnosed with TBI.^3^ TBIs are commonly divided into mild, moderate, severe, and penetrating. Most are mild, comprising ∼85% of all TBIs.^2^ The recorded incidence of mild TBI (mTBI) among U.S. military SMs has increased over the past 20 years, due in part to increased screening and awareness. Acute screening and evaluation of mTBI were enhanced in the deployed environment through Department of Defense (DoD) Instruction 6490.11,^4^ mandating screening and reporting of TBI injuries among military SMs as of September 18, 2012.

Most individuals who experience mTBI typically recover within 3 months of injury.^5,6^ However, for a minority, symptoms persist. Much research has attempted to identify the factors that predict who will not recover. One potential risk factor is concurrent extra-cranial bodily injuries. Some research has shown that the presence of significant bodily injuries, or polytrauma, leads to poorer functional recovery at 3 and 6 months after TBI.^7^ In contrast, when the relationship between bodily injury and recovery from mTBI was tested in war-era military SMs with deployment injuries evaluated at a range of times postinjury,^8,9^ the presence of concurrent bodily injuries was associated with lower postconcussive symptom scores on the Neurobehavioral Symptom Inventory (NSI).^10^

Several possible explanations were proposed for the lower NSI scores among the SMs with concurrent bodily injuries, including (1) underreporting postconcussive symptoms when other injuries and associated pain are a focus of treatment and attention (or elevating symptom report when an “invisible” mTBI is the only injury), (2) receiving increased interpersonal and medical support associated with treatment of the bodily injuries, (3) experiencing the effects of morphine and other opioids, and (4) delaying endorsement of postconcussive symptoms until after treatment and healing of the bodily injuries.

Given that these studies were conducted on SMs who received blast injuries and/or were deployed to combat theaters in Iraq and Afghanistan during the post-9/11 era, the presence of psychological trauma likely influenced the results. It is well established that combat deployment is associated with an increased risk for the development of acute stress and post-traumatic stress disorder (PTSD).^11^ A history of mTBI also increases the risk for stress disorders among deployed SMs.^12^ Multiple studies confirm the relationship between self-reported postconcussive symptom severity and the presence and severity of PTSD.^13,14^ However, differences in PTSD incidence and severity following mTBIs with other concurrent bodily injuries, versus those without, have not been well characterized and are examined in the current study.

### Under- or overreporting symptoms

A challenge related to the research and clinical care of patients with a history of mTBI is concern about the validity of self-reported injuries among SMs. Reluctance to appear weak or injured can lessen reported symptoms and symptom severity of an injury or even lead to denial of the injury. In contrast, an SMs may overestimate the severity of symptoms due to distress or a desire to express the subjective reality of the symptoms to providers and others. The latter is particularly likely when an SM has an “invisible” injury, such as mTBI.^14^

Hypothesis 1: Postconcussive symptom severity is lower when bodily injuries direct focus and attention away from TBI-specific symptoms. More pain and distress from bodily injuries detract from the identification and endorsement of postconcussive symptoms. In contrast, SMs with mTBI and no associated bodily injuries are more likely to enhance or overemphasize their postconcussive symptoms to correspond with their subjective distress.

### Interpersonal support

It is known that frequent positive interpersonal support aids recovery from both bodily injuries^15^ and TBI.^16^ Bodily injuries often require acute treatment and subsequent therapy associated with interpersonal contact and support for the patient. SMs with bodily injuries frequently engage in physical rehabilitation in an inpatient or outpatient setting. In a home environment, they often need someone to assist them with wound care and/or daily activities. Professional interactions with the patient’s family during the healing process tend to increase family knowledge about the injury and elicit their greater support and care. In contrast, if an SM has mTBI with no bodily injuries, treatment might not be provided at all. If it is, it will almost always be on an outpatient basis for a limited time.

Hypothesis 2: SMs with a history of mTBI and associated bodily injury will receive more interpersonal support from medical staff and family/friends, which will be associated with lower self-reported postconcussive symptoms.

### Morphine and opioid analgesics

The Department of Veterans Affairs (VA) and DoD clinical practice guidelines for both the management of opioid therapy^17^ and mTBI^18,19^ caution against using opioids as first-line therapy in individuals with mTBI. This is due to the well-documented negative cognitive effects of opioids, as well as the relationship between opioids, mental health conditions, and substance use disorders, increasing the risk for serious adverse clinical outcomes, including opioid overdose. It is of note that although opioid therapy is contraindicated in patients with mTBI, implementation of the guideline is left to clinical judgment in each specific case, and opioid medication is known to sometimes be prescribed to SMs after mTBI.^20^ According to a recent scoping review,^21^ presence of pain and psychological conditions such as depression and PTSD among individuals with a history of TBI are associated with receiving opioid prescriptions. Veterans with TBI, PTSD, and a chronic pain diagnosis (i.e., “polytrauma clinical triad”)^13^ receive the highest rate of prescription opioids compared with veterans with fewer of these conditions.

Opioids inhibit pain and fear and reduce avoidance behaviors.^22^ A study by Melloney^23^ examining fear avoidance in subjects with TBI, of whom most were mTBI, found that catastrophizing and fear avoidance behavior correlated significantly with elevated postconcussive symptoms (*p* < 0.05). In another study, Silverberg^24^ examined fear avoidance in relation to several outcome measures, including postconcussive symptoms, functional disability, return to work, and psychiatric conditions. Fear avoidance was significantly related to poor symptom outcome, disability, and anxiety. Although current clinical guidelines recommend avoiding opioids following mTBI due to their addictive and overdose potential and their negative effects on cognitive function,^19^ these medications also have positive effects in reducing fear avoidance and improving symptomatic and functional outcomes after mTBI.

Hypothesis 3: Comparing narcotic medication use for the two groups, the group with bodily injuries will have a higher proportion of SMs taking these medications. In addition, taking opioids will be associated with lower postconcussive symptom scores.

### Delayed expression of symptoms

Most patients with a history of mTBI follow a course of recovery in which acute symptoms resolve quickly, at most within 3 months of injury.^5,6^ Delayed symptoms are often associated with other illnesses or injuries, situational stressors, sleep disorders, chronic pain, substance use disorders, adverse events, and psychological distress.^13^ SMs with bodily injuries are theorized to report more postconcussive symptoms when their attention is shifted from physical injuries to their postconcussive cognitive and emotional functioning. At later times after injury, SMs are generally more engaged in challenging activities as they attempt to return to their prior level of function and productivity. This is a time of vulnerability for increased symptom expression as SMs become more aware of any residual weaknesses and limitations. Among SMs with bodily injuries, this time occurs after the time required to recover from the bodily injury.

Hypothesis 4: Delayed symptoms occur due to increased mental, emotional, and physical demands as SMs attempt to return to their prior level of functioning. Among SMs with bodily injuries, this time occurs once the bodily injury has healed. Participants in the bodily injury group evaluated later after injury are therefore predicted to report more postconcussive symptoms than those evaluated earlier. A positive association is expected between time since injury (TSI) and the NSI total score for the group with bodily injuries. In contrast, the group without bodily injuries is expected to show a less time-dependent change in postconcussive symptom scores.

## Materials and Methods

### Participants

Demographic, injury-related, and symptom severity data from 165 active-duty SMs and veterans were extracted from a large TBI data registry housed at a major military medical center. All participants included in the registry were recruited from primary care clinics affiliated with the medical center. Participants in this study were evaluated and enrolled in the registry between January 1, 2017, and May 31, 2018. The inclusion criteria for the study were (1) meeting the standard VA/DoD criteria for mild TBI,^19^ (2) having scores coded on the Abbreviated Injury Scale (AIS), and (3) completing the NSI.

### Measures

#### Bodily injury

The AIS^25^ was used as the measure of bodily injury. It is based on an anatomical model classifying each injury by one of six body regions: (1) head/neck/C-spine, (2) face, (3) thorax/T-spine, (4) abdomen/L-spine, (5) extremities, and (6) external/burns. Each injury is coded for its specific location and type and is rated for severity on a 6-point scale. For this study, participants with one or more injury code(s) in areas 3 through 6 were included in the bodily injury group (*n* = 48), whereas those with no injury codes in areas 3 through 6 were placed in the no bodily injury group for analyses (*n* = 117). Only extra-cranial injuries were included, omitting any injuries coded in region 1. In addition, injury region 2 (face) was also omitted from the classification because there were only two codes used for this area in the sample, corresponding to “headache” and “tinnitus.” These two codes were considered symptoms of TBI rather than injuries received.

#### Postconcussive symptom severity

A total score on the NSI^10^ was used as a measure of postconcussive symptom severity. The NSI is a 22-item self-report measure of postconcussive symptoms. Each item is rated on a 5-point scale from 0 (none; symptom is rarely ever present/not a problem at all) to 4 (very severe; symptom is almost always present/impairs performance at work, school, or home/individual probably cannot function without help). The NSI has been shown to be a reliable and valid measure of postconcussive symptoms in veterans.^26^ It is highly correlated with emotional distress, including PTSD, depression, and anxiety, among military and veteran populations.^27^

#### Post-traumatic symptom severity

The measure of post-traumatic symptom severity at evaluation was the total score on the PTSD Checklist, Civilian Version (PCL-C).^28,29^ The military version was not used because it specifically queries for symptoms associated with military trauma. We chose the Civilian Version to encompass all potential sources of trauma. The PCL-C is a 17-item self-report measure of diagnostically relevant PTSD symptoms. Each item is rated on a 5-point scale from 1 (not at all) to 5 (extremely). The PCL-C is a commonly administered, reliable, and valid measure of PTSD symptoms in veterans.^30^ It is correlated with postconcussive symptoms among individuals with a history of TBI.^31^

#### Pain

Score on Part A of the McGill Pain Questionnaire—Short Form (MPQ-SF)^32^ was defined as the measure of bodily pain. This score is based on a visual analog scale with a range from 0 to 99, with higher scores representing more pain.

#### Overestimation of symptoms

Postconcussive symptom overreporting was defined by a score on the Validity-10 scale of the NSI. The Validity-10 scale score ranges from 0 to 40, with each of the 10 items scored from 0 to 4. It has been shown to have clinical utility in measuring symptom overreporting among military SMs.^33,34^ A total score of 23 is the optimal cut point for invalidity on the NSI items that are infrequently endorsed by individuals with TBI, according to the original Validity-10 development study.^35^ Overreporting in the current study was defined as a score greater than +1 standard deviation (*sd*) above the mean on the Validity-10 scale (≥18) and less than or equal to the maximum valid score of 22, as reported in the original development study.^35^

#### Social support

The social support item (item #2) from the original 25-item Connor Davidson Resilience Scale (CD-RISC),^36^ ‘I have at least one close and secure relationship that helps me when I am stressed,’ was compared for the two groups to assess the level of social support. Item scores range from 0 to 4 with higher scores representing more frequent support (0 = not true at all; 1 = rarely true; 2 = sometimes true; 3 = often true; 4 = true nearly all the time).

#### Narcotics use

The use of narcotic medications was recorded from the date of injury to the date of evaluation. The date of injury was obtained from self-report and, where available, verified with a review of electronic medical records. Participants who were administered morphine or other opioid narcotics acutely after injury, at any time since the injury, or at evaluation were considered “positive” for narcotic medication administration.

#### Time since injury

The number of months from the date of injury to the date of evaluation was calculated as TSI.

### Procedures

A local TBI registry stored on a restricted network drive in a major military medical center was the source of data for this study. Registry variables were obtained by self-report and semi-structured interview conducted by a registered nurse researcher. The registry was approved by the institutional review board of the medical center. At the time of enrollment, each participant signed written consent for the storage and release of their information for future research.

Demographic, TBI injury, and study variables were extracted from the registry, verified by review of the medical center’s electronic medical records, and compiled in an SPSS version 22 deidentified database (Tables 1 and 2). The study was completed in accordance with the guidelines of the Declaration of Helsinki and was undertaken with the understanding and written consent of participants.

**Table 1.** Demographic Characteristics for the mTBI Groups with Bodily Injury and Without Bodily Injury

	Bodily injury (*n* = 48) *n* (%)	No bodily injury (*n* = 117) *n* (%)	χ^2^ (*df*)	*p*
Gender			3.82 (1)	**0.05**
Male	37 (77%)	104 (89%)		
Female	11 (23%)	13 (11%)		
Race			0.64 (3)	0.89
White	22 (46%)	61 (52%)		
Hispanic	13 (27%)	26 (22%)		
Black	10 (21%)	23 (20%)		
Asian/Mixed/Other	3 (6%)	7 (6%)		
Marital			0.89 (1)	0.34
Married/Cohabiting	34 (71%)	91 (78%)		
Single/Divorced/Sep/Widow	14 (29%)	26 (22%)		
Education (years)			2.83 (3)	0.42
<12	5 (10%)	5 (4%)		
13–14	25 (52%)	58 (50%)		
15–16	13 (27%)	37 (32%)		
>16	5 (10%)	17 (14%)		
Age (years)	**Mean (*sd*)**	**Mean (*sd*)**	***t (df)***	**p* (t)***
	36.1 (7.9)	37.7 (6.6)	1.36 (163)	0.18

*df*, degrees of freedom; mTBI, mild traumatic brain injury; *n*, sample size; *p*, two-sided probability value.

**Table 2.** mTBI Injury Characteristics for the mTBI Groups with Bodily Injury and Without Bodily Injury

	Bodily injury (*n* = 48) *n* (%)	No bodily injury (*n* = 117) *n* (%)	χ^2^ (*df*)	*p*
Loss of consciousness	*n* = 43	*n* = 108	**6.76 (2)**	**0.034**
None	18 (42%)	67 (62%)		
<1 min	9 (21%)	21 (19%)		
1 min or longer	16 (37%)	20 (19%)		
Post-traumatic amnesia	*n* = 41	*n* = 111	**14.54 (3)**	**0.002**
None	13 (32%)	65 (59%)		
<1 min	6 (15%)	20 (18%)		
1–15 min	11 (27%)	17 (15%)		
>16 min	11 (27%)	9 (8%)		
Injury location	*n* = 48	*n* = 116	**5.33 (1)**	**0.021**
War zone (OIF/OEF/OND/GWOT)	13 (27%)	54 (47%)		
Nonwar zone (CONUS/non-CONUS)	35 (73%)	62 (53%)		
Injury mechanism	*n* = 48	*n* = 115	**13.13 (1)**	**<0.001**
Blast	5 (10%)	45 (39%)		
Nonblast	43 (90%)	70 (61%)		
Injury type	*n* = 48	*n* = 117	**13.28 (1)**	**<0.001**
Combat	8 (17%)	55 (47%)		
Noncombat	40 (83%)	62 (53%)		
Number of deployments	*n* = 48	*n* = 116	5.42 (3)	0.14
0	9 (19%)	9 (8%)		
1	7 (15%)	29 (25%)		
2–3	24 (50%)	59 (51%)		
4 or more	8 (17%)	19 (16%)		

*df*, degrees of freedom; mTBI, mild traumatic brain injury; *n*, sample size; *p*, two-sided probability value.

### Statistical analyses

The relationship between postconcussive symptom severity and the presence or absence of associated bodily injury was examined with an independent samples *t*-test comparing the mean total NSI score for the group with bodily injuries with the group with no bodily injuries. Group differences on categorical background and injury-related variables (e.g., gender, marital status, and mechanism of injury) were compared with χ^2^ tests. Differences on ordinal and interval-level variables were compared with independent groups *t*-tests.

The four major hypotheses were tested by comparing group scores on each factor theorized to account for postconcussive symptom differences between groups. Independent groups *t*-tests compared mean group scores on hypothesized factors of pain, social support, and delayed symptoms. Chi-squared tests compared the percentage of each group overreporting postconcussive symptoms and using narcotics. Each factor score was also correlated with the total NSI score for each group.

For hypothesis 1, pain scores on the MPQ-SF were compared for the two groups and correlated with mean total NSI scores for each group. It was predicted that pain scores for the group with a bodily injury would be higher and would correlate negatively with postconcussive symptom severity. A second part of hypothesis 1 predicted that the group with no bodily injuries would overreport postconcussive symptoms to express their distress associated with the “invisible” mTBI. This hypothesis was tested by calculating and comparing the percentage of SMs in each group scoring 18–22 (“overreporting”) on the Validity-10 scale from the NSI. It was predicted that proportionately more of the group with no bodily injuries would have NSI scores in the “overreporting” range.

To test hypothesis 2, social support scores on CD-RISC item 2 were compared across groups and correlated with total NSI scores for each of the two groups. It was predicted that the group with bodily injury would have higher social support scores, which would correlate negatively with postconcussive symptom severity, as measured by total NSI scores. These results would indicate that the group with bodily injuries has more social support, and the higher the support, the lower the postconcussive symptom severity compared with the group with no bodily injuries.

Hypothesis 3 was tested by calculating and comparing with χ^2^ the percentage of SMs in each group with narcotic use. It was predicted that proportionately more of the group with bodily injuries would have narcotic use, and NSI scores would be lower for those with narcotic use.

Hypothesis 4 was tested by correlating TSI with total NSI scores for each of the two groups. It was predicted that for the group with a bodily injury, TSI would correlate positively with postconcussive symptom severity. That is, as time increased after injury, more delayed postconcussive symptoms would be endorsed. Delayed postconcussive symptoms were predicted to occur once the pain associated with a bodily injury was resolved. To better approximate this possibility, TSI for each individual was classified as either “short” or “long,” based on a mean TSI split for each injury group. Analysis of variance (ANOVA) on total NSI scores tested for main effects of TSI (short or long) and participant group (with or without bodily injuries). A significant interaction effect was predicted, with lower symptom scores for short TSI and higher symptom scores for long TSI, in the group with bodily injuries.

Hierarchical multiple regression was used to predict the NSI score from the injury group and the four hypothesized explanatory factors (pain, social support, narcotics use, and delayed symptoms). Separate analyses predicted NSI scores for each injury group, controlling for covariates. Covariates were defined as background and injury variables differing across groups (i.e., gender, duration of loss of consciousness [LOC], duration of post-traumatic amnesia [PTA], blast/nonblast, combat/noncombat injury, and war zone/nonwar zone injury location). In a separate regression analysis, PCL-C score was included as an additional covariate.

## Results

### Sample characteristics

Basic demographic and TBI-related data for the sample are shown in Tables 1 and 2. In this sample, the group with concurrent bodily injuries comprised of proportionately more females compared with the group without bodily injuries. As presented in Table 1, no other demographic characteristic differed significantly between the two groups. There were several significant differences between groups on injury-related characteristics, including the severity of mTBI indices (LOC and PTA). The group with bodily injuries comprised of proportionately more SMs with more severe mTBIs, as indicated by the presence and duration of LOC and PTA, as shown in Table 2. In this sample, participants with bodily injuries were more likely to have been injured in a nonwar zone, in noncombat, and from a mechanism other than blast.

### Symptom differences between groups

As shown in Table 3, the mean total symptom score on the NSI for the mTBI plus bodily injury group was lower but not significantly lower than the mean total score for the mTBI without bodily injuries group. The effect size was low (*d* = 0.05), reflecting the large variability and overlap in scores across the two groups. Covarying the demographic and injury-related characteristics that differed between groups (gender, duration of LOC and PTA, blast, combat, and war-zone injuries) resulted in a similar nonsignificant difference between groups on total NSI score (bodily injury group adjusted mean NSI = 33.6, *sd* = 21.3; no bodily injury group adjusted mean NSI = 34.4, *sd* = 20.5; *F* = 0.21, degrees of freedom [*df*] = 1,142, *p* = 0.65). Due to missing data, the analysis with covariates included 40 of the 48 (83%) participants with bodily injuries and 103 of the 117 (88%) participants with no bodily injury.

**Table 3.** Postconcussive and Post-Traumatic Symptom Scores for the mTBI Groups with Bodily Injury and Without Bodily Injury

	Bodily injury (*n* = 48) Mean (sd)	No bodily injury (*n* = 117) Mean (sd)	t (df)	*p*
Postconcussive symptoms (NSI total score)	34.2 (19.8)	35.3 (21.0)	0.31 (163)	0.38
Post-traumatic stress symptoms (PCL total score)	42.7 (17.9)	45.6 (20.0)	0.88 (163)	0.19

*df*, degrees of freedom; mTBI, mild traumatic brain injury; *n*, sample size; NSI; Neurobehavioral Symptom Inventory; *p*, one-sided probability value; *sd*, standard deviation.

Mean symptom scores on the PCL-C for the two injury groups are also shown in Table 3. These results parallel those found for NSI scores, with nonsignificantly lower PCL-C scores for the group with bodily injury. The effect size was low (*d* = 0.15). Analysis with covariates also resulted in a nonsignificant difference between groups on the total PCL-C score (bodily injury group adjusted mean PCL-C = 42.5, *sd* = 19.1; no bodily injury group adjusted mean PCL-C = 45.2, *sd* = 20.2; *F* = 0.85, *df* = 1,142, *p* = 0.36).

### Predictive factors for postconcussive symptoms

#### Hypothesis 1a: Pain/underreporting symptoms

The average pain severity rating (range = 0–99) on the MPQ-SF for the group with bodily injuries was 44.1 (*sd* = 31.8), whereas the mean score for the group with no bodily injuries was 38.2 (*sd* = 30.4) (*t* = 1.12, *p* = 0.13; Cohen’s *d* = 0.19). The Pearson correlation between pain rating and NSI total score was 0.47 (*p* < 0.001). The correlation was 0.37 (*p* = 0.01) for the group with bodily injuries and 0.52 (*p* < 0.001) for the group with no bodily injuries. These results indicate that subjective pain was not significantly different for the two groups. Both groups’ pain levels were significantly positively correlated with their postconcussive symptom severity scores, with a somewhat higher correlation for the group with no bodily injuries.

#### Hypothesis 1b: Overreporting symptoms

For the group with bodily injuries, 3 of 48 (6.2%) participants overestimated the severity of postconcussive symptoms using Validity-10 scores without reaching the level where validity would be questioned. For the group with no bodily injuries, a similar proportion (8 of 117; 6.8%) overestimated symptoms. Among those with bodily injuries, the mean Validity-10 score was 11.4 (*sd* = 8.3), whereas the mean score was 11.8 (*sd* = 9.3) for those with no bodily injuries (*t* = 0.20, *df* = 163, *p* = 0.42). Based on these results, in contrast to our hypothesis, SMs with no bodily injuries did not overestimate postconcussive symptoms compared with SMs with bodily injuries.

#### Hypothesis 2: Social support

When item 2 from the original 25-item CD-RISC was compared for the two groups as a measure of social support, the group with injuries had a mean social support score of 3.3 (*sd* = 1.1) compared with 3.3 (*sd* = 1.0) for the group without injuries (*t* = −0.29, *df* = 150, *p* = 0.39). Thus, perceived social support did not differ across the bodily injury groups. Social support was, however, significantly negatively related to postconcussive symptom severity as measured by the total score on the NSI for the total sample (Pearson *r* = −0.20, *n* = 152, *p* = 0.01). Correlations for the two injury groups separately showed trends toward significance (mTBI plus bodily injury group: Pearson *r* = −0.27, *n* = 48, *p* = 0.07 and mTBI without bodily injury group: Pearson *r* = −0.17, *n* = 104, *p* = 0.09). The negative correlations indicate that more perceived social support is associated with lower postconcussive symptom scores.

#### Hypothesis 3: Opioid/narcotic use

Of the 145 participants with medication data, 14 were prescribed morphine or narcotics after their injury. Five of the 14 had bodily injuries, whereas 9 did not. The proportion of the bodily injury group taking narcotics (5/41 = 9.8%) was not significantly different than the proportion of the group without bodily injuries taking narcotics (9/104 = 8.7%) (χ^2^ = 0.04, *df* = 1, *p* = 0.83). Therefore, the use of narcotic medication was not selectively associated with the presence of bodily injuries in this sample.

For the group with bodily injuries, those taking narcotics (*n* = 5) had a mean total NSI score of 30.8 (*sd* = 24.1). For the group with no bodily injuries, those taking narcotics (*n* = 9) had a mean total NSI score of 41.0 (*sd* = 22.5). The latter result suggests that the use of narcotics by those with no associated bodily injuries may be deleterious to symptomatic recovery or, alternatively, that those with more symptoms are more likely to be administered narcotic medication. Due to small sample sizes, the scores were not significantly different for the two groups despite apparent mean differences (*t* = 0.79, *df* = 12, *p* = 0.44). The effect size was medium (Hedge’s *g* = 0.41).

Examination of PCL-C scores revealed similar findings in that those taking narcotics in the group with bodily injuries (*n* = 5) had a mean total PCL-C score of 38.2 (*sd* = 16.9). Those taking narcotics in the group with no bodily injuries (*n* = 9) had a mean total PCL score of 51.4 (*sd* = 20.3). These scores did not significantly differ for the two groups (*t* = 1.2, *df* = 12, *p* = 0.24), but the effect size was medium (Hedge’s *g* = 0.64).

#### Hypothesis 4: Delayed symptom expression

The time interval between the date of injury and the date of evaluation varied from 6 days to 28.4 years. Most of the sample was in the chronic phase of mTBI recovery, having been evaluated more than 6 months after injury. The mean time from mTBI to evaluation (TSI) for the group with bodily injuries was 49.8 months (*sd* = 62.1), or ∼4 years, and the mean time for the group with no bodily injuries was significantly greater, at 74.0 months (*sd* = 55.5) or 6.2 years (*t* = 2.44, *df* = 161, *p* = 0.016).

**FIG. 1.**
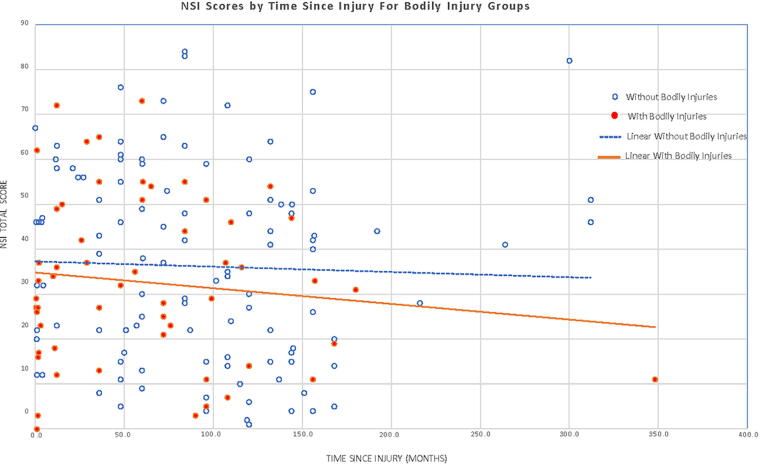
NSI scores and time since injury for bodily injury groups. NSI, Neurobehavioral Symptom Inventory.

Figure 1 presents NSI scores as a function of the number of months from injury to evaluation for participants in the two groups. The linear regression lines illustrate the similar NSI scores across time for those in the group with bodily injuries compared with the group with no bodily injuries. Correlations between time postinjury and NSI scores were not significant for the total sample (*r* = −0.10, *p* = 0.22) or for the groups with bodily injuries (*r* = −0.16, *p* = 0.28) and with no bodily injuries (*r* = −0.08, *p* = 0.38). When TSI was categorized as either “early” or “late” based on a mean split of TSI for each group, there was again no evidence for a delayed increase in scores in the injury group. A two-way ANOVA yielded no significant effects for the injury group (*F* = 0.26, *p* = 0.61), early versus late time of evaluation (*F* = 1.3, *p* = 0.26), or the interaction (*F* = 0.11, *p* = 0.74).

### Prediction of NSI scores

Hierarchical multiple regression was conducted with covariates loaded in block 1 and subsequent stepwise entry of the injury group and the four predictor variables of pain, social support, use of narcotic medication, and TSI on the total NSI scores for the full sample. Demographic and mTBI injury-related characteristics with differences between bodily injury groups were treated as covariates entered in block 1 (i.e., gender, duration of LOC, duration of PTA, blast/nonblast, combat/noncombat injury, and war zone/nonwar zone injury location). Pain rating was the first predictor variable to enter the model (*β* = 0.44, *t* = 5.3, *p* < 0.001) and social support was entered next (*β* = −0.21, *t* = −2.6, *p* = 0.01). The direction of these associations indicates that the higher the individual’s pain level, the more postconcussive symptoms were endorsed. The more the social support, the fewer the symptoms endorsed. The other predictive factors, use of narcotics, TSI, and injury group were not significantly related to the total NSI score once pain and social support were entered into the model.

Regression analyses were also conducted to predict NSI scores based on the four predictor variables for the two injury groups separately. For the group with bodily injuries, pain was the only significant predictor (*β* = 0.33, *t* = 2.28, *p* = 0.03), whereas, for the group with no bodily injuries, pain (*β* = 0.54, *t* = 6.28, *p* < 0.001) and social support (*β* = −0.18, *t* = −2.11, *p* = 0.037) were significant predictors of NSI scores.

### Inclusion of PCL-C score as a covariate

A separate regression was conducted including the PCL-C score as another covariate in the model. For the total sample with nonmissing variables (*n* = 126), pain rating was the first and only variable to enter the model (*β* = 0.12, *t* = 2.95, *p* = 0.004). Like results without PCL-C included as a covariate, this analysis indicated that the higher the pain level, the higher the NSI score, after accounting for group differences in demographic, injury-related variables, and post-traumatic stress symptoms. The other three predictors, social support, use of narcotics, and TSI, were not significantly related to the NSI total score when PTSD symptom score was included as a covariate in the model.

## Discussion

The major analyses of this study examined factors that might have contributed to the results of prior studies^8,9^ revealing lower postconcussive symptom scores among SMs with mTBI and concurrent bodily injuries compared with those with mTBI and no concurrent bodily injuries. Present results assessed the generalizability of prior results to a more recently evaluated, more heterogeneous sample of SMs with a history of mTBI. Findings did not reach the level of statistical difference in this more recent sample, although the direction of group differences concurred with previous findings showing that postconcussive symptom scores on the NSI tend to be lower for SMs with mTBI and concurrent bodily injuries than for SMs with mTBI and no concurrent bodily injuries.

When this effect was initially reported,^8^ it was theorized that the findings were due to overreporting of symptoms by some participants with mTBI without bodily injury.^8^ Upon replication of findings in a separate sample,^9^ four other possible mechanisms were proposed to explain these counterintuitive findings. When these mechanisms were examined in the current study, the results indicated that none showed significant between-group differences. However, pain and social support were significantly correlated with postconcussive symptom scores on the NSI. As measured in this study, pain was positively correlated with NSI at a significant level, whereas perceived social support was negatively correlated with it. This is consistent with research that has shown the negative effects of pain and the beneficial effects of interpersonal and social support on symptomatic mTBI recovery.^16,37^ The relationship between pain and NSI was significant for the total sample, as well as for both participant groups individually. Although social support was significantly correlated with scores on NSI for the total sample, the correlations for the bodily injury groups separately showed trends toward significance.

The third hypothesis of this study predicted that narcotics would be prescribed and used more frequently among those with bodily injuries, lowering their postconcussive symptom scores and improving outcomes. Results indicate that the proportion of participants using narcotics was equivalent for the two groups. Postconcussive symptom severity was somewhat but not significantly lower in the bodily injury group among those administered or prescribed narcotics. In contrast, symptom severity was highest among SMs with no bodily injuries for whom narcotics were administered. Unfortunately, information indicating the reasons narcotics were prescribed and the duration of use and dosage was not available for this study. However, research suggests that narcotics are primarily prescribed and used by individuals with mTBI due to complaints of emotional distress or psychological symptoms of comorbid depression, anxiety, or PTSD.^21^ Based on this information, we conducted a *post hoc* comparison of PCL-C scores for those taking narcotics (*n* = 14, mean = 46.7, *sd* = 19.6) and those not taking narcotics (*n* = 144, mean = 44.5, *sd* = 19.6) (*t* = −0.40, *df* = 156, *p* = 0.69). The mean score on PCL-C for those prescribed narcotics was not significantly different than the mean PCL-C score for those that were not. Results were therefore not consistent with research indicating that narcotics are used more frequently among patients who endorse high levels of psychological symptoms, at least as reflected by PCL-C.^21,38^ The design of the current study, utilizing a cohort of participants evaluated at a single time point, is not able to address questions about the timing of symptoms relative to narcotic use. Many of the participants might have taken narcotics early in their recovery, which could have been up to years prior to their evaluation. In addition, data on narcotics use were obtained from self-report at evaluation and review of available medical records from the time of injury to evaluation, which was subject to recall bias and incomplete documentation.

Other reasons for prescribing narcotics include the presence of pain and the elevated severity of postconcussive symptoms.^39^ Differences in NSI and PCL-C scores between groups prescribed and not prescribed narcotics were not statistically significant in the present study, perhaps partly due to the timing of the evaluation during the chronic phase of mTBI recovery and the low numbers of participants reporting that narcotics were used or prescribed for their injuries.

A fourth hypothesis tested in this study proposed that elevations in NSI scores among those with bodily injury are delayed after injury. Examination of individual trajectories of recovery over TSI was not possible in this cross-sectional study sample with a single evaluation point. However, participants were evaluated over a wide range of time post-injury. Results showed that patients with bodily injuries who were evaluated later after injury tended to rate their postconcussive symptoms as less severe than those evaluated sooner after injury. SMs without bodily injuries rated their symptoms at an overall consistent level, regardless of the time since the injury.

In summary, the results of between-group comparisons did not support the four hypothesized reasons for a lower postconcussive symptom report among SMs with mTBI and bodily injuries. However, the results of correlational and multiple linear regression models suggest that postconcussive severity scores among SMs with mTBI and bodily injuries are highly related to perceived level of pain. Among those with mTBI and no bodily injuries, postconcussive severity scores are also related to subjective pain level as well as secondarily to perceived social support.

Several sample differences between the present study and past studies might account for differing results. In this study, proportionately more females were included in the group with bodily injuries. Females report more symptoms and a longer recovery.^40^ In the general population, pain, anxiety, and PTSD are more common in women. Thus, the mean NSI score of the group with injuries would be expected to be higher due to a higher proportion of females. An elevation in the mean NSI score of the group with bodily injuries would reduce the difference between groups, as seen in the present results.

Other differences between the current and prior studies include the mechanism, location, and environment of injury. Earlier studies were conducted with war-era SM that received injuries during combat deployment. In contrast, the present study included SM with any injury mechanism received in any location. In the current sample, the group with bodily injuries was composed mainly of nonblast, noncombat, and nonwar zone injuries. Considering these characteristics, lower NSI scores in this group are perhaps not surprising. Among the group with no bodily injuries, other emotional and mental health conditions might be acting as sources of elevation in postconcussive symptom scores. In opposition to this theory, groups did not significantly differ in the level of post-traumatic stress symptoms. In addition, the results of the regression analyses did not differ when the PCL-C score was included as a covariate.

It should be noted that the bodily injuries associated with mTBI in the present sample were relatively mild. Among the group with bodily injuries (*n* = 48), there were only four participants with a modified Injury Severity Score (ISS_mod_) greater than 15, a common criterion for major trauma or polytrauma. In the earlier French study,^9^ 23.4% of participants had polytrauma, and their symptom ratings were significantly lower than the other participants with milder bodily injuries.

Another important factor that was not assessed in this study was the attribution of the symptom report. SM may perceive a symptom as related to the mTBI or to another factor such as PTSD, depression, stress, or an associated bodily injury. The total score on the NSI will be lower when the respondent perceives fewer symptoms to be caused by mTBI.

## Conclusions

In summary, the results of this study suggest that the level of subjective pain is a determinant of postconcussive symptom severity among SMs with a history of mTBI, with or without associated bodily injuries. Social support is a weaker secondary predictor of lower levels of postconcussive symptoms following mTBI among SMs with no associated bodily injuries. Relating these results to clinical care, SMs with a history of mTBI are expected to benefit symptomatically from good control of their subjective level of pain. Among those with mTBI and no associated bodily injuries, social support also plays a role in reducing postconcussive symptoms.

## Disclaimer

The views expressed in this article are those of the authors and do not necessarily represent the official policy or position of the Defense Health Agency, Department of Defense, or any other U.S. government agency. This work was prepared under Contract HT0014-22-C-0016 with DHA Contracting Office (CO-NCR) HT0014 and, therefore, is defined as U.S. government work under Title 17 U.S.C.§101. Per Title 17 U.S.C.§105, copyright protection is not available for any work of the U.S. government. For more information, please contact dha.TBICOEinfo@health.mil. UNCLASSIFIED.

## Notes

### Abbreviations Used

AIS: Abbreviated Injury Scale
ANOVA: Analysis of variance
CD-RISC: Connor Davidson Resilience Scale
CONUS: Contiguous States
DoD: Department of Defense
GWOT: Global War on Terrorism
ISS MOD: Modified Injury Severity Score
LOC: Loss of consciousness
MPQ-SF: McGill Pain Questionnaire-Short Form
mTBI: Mild traumatic Brain injury
Non-CONUS: Outside Contiguous States
NSI: Neurobehavioral Symptom Inventory
OEF: Operation Enduring Freedom
OIF: Operation Iraqi Freedom
OND: Operation New Dawn
PCL-C: PTSD Checklist-Civilian Version
PTA: Post Traumatic Amnesia
PTSD: Post Traumatic Stress Disorder
SD: Standard deviation
SMs: Service Members
TBI: Traumatic Brain Injury
TSI: Time since injury
VA: Department of Veterans Affairs
